# Accurate localization of life threatening colonic hemorrhage during nuclear medicine bleeding scan as an aid to selective angiography

**DOI:** 10.1186/1749-7922-4-20

**Published:** 2009-05-27

**Authors:** Mubin I Syed, Azim Shaikh

**Affiliations:** 1 Wright State University School of Medicine, Dept of Radiological Sciences, Dayton, Ohio, USA; 2 Dayton Interventional Radiology, 3075 Governors Place Blvd, Suite 120 Dayton, Ohio 45409, USA; 3 Department of Interventional Radiology, Springfield Regional Medical Center, 2615 East High St, Springfield, Ohio 45504, USA

## Abstract

**Purpose:**

To describe a new technique to help localize life threatening colorectal bleeding during nuclear medicine bleeding scan to aid in selective angiography.

**Methods:**

During the gastrointestinal bleeding scan, a simple metallic marker (paper clip) was used to localize the bleeding site on the patient body. Angiography was then performed within 2 hours. The marker was then used to guide superselective angiography and embolization.

**Results:**

5 cases of patients with colorectal bleeding were performed using this technique with cessation of bleeding in 4/5 initial attempts. 1 patient required a repeat angiogram that did demonstrate the bleeding on the second attempt allowing superselective angiography and embolization that resulted in cessation of bleeding. This patient with a rectal bleed required selection of additional vessels guided by the marker on the second attempt.

**Conclusion:**

The dilemma of positive scintigraphic evidence of colonic bleeding with negative arteriography can be resolved with the use of a metal marker during the scintigram to guide superselective angiography. Although in our small series of patients this technique appears to be simple and effective, further clinical investigation is warranted with a larger patient population. This technique may offer a role in therapy in coordination with the colorectal surgeon for the high risk patient in an otherwise life threatening situation.

## Introduction

Gastrointestinal hemorrhage is a life-threatening situation with up to a 10% mortality rate when emergent surgery is performed. [1] Localization of the hemorrhage by a nuclear medicine scan is a useful first step for treatment with endoscopy, surgery, and/or by catheter directed embolization. Embolization has gained widespread acceptance for the treatment of upper gastrointestinal hemorrhage and more recently for lower gastrointestinal hemorrhage. The limitation of the technique has always been the lack of the active bleeding during arteriography despite active bleed on the nuclear medicine scan. This can be due to the intermittent nature of gastrointestinal bleed as well as the discrepancy in sensitivity between angiography and the nuclear scan. The nuclear scan is significantly more sensitive for bleeding then angiography, which can only detect bleeding at rate of 0.5 cc/minute. We present a simple technique for localization of colonic bleed seen on the bleeding scan even if not visible with initial angiography that may guide superselective arteriography.

## Methods

Institutional Review Board approval was obtained for a retrospective review. Between 1999 and 2007 a total of 5 patients with colonic bleeding underwent localization using the technique described below.

### Localization of hemorrhage on nuclear medicine bleeding scan

During the gastrointestinal bleeding scan, a simple metallic marker (paper clip) was used to localize the bleeding site on the patient's body. A standard nuclear medicine scintigram is performed using Tc-99^m ^tagged red blood cells. The site of bleeding is visualized and identified on the image monitor. While the patient is still under the gamma camera, a small 10 millimeter diameter cobalt-57 marker is placed directly on the patient's skin over the identified bleeding site (using the image monitor for guidance). The radioactive source should be placed immediately when extravasation is identified either during the early flow phase of the study or the subsequent five minute static images depending on rate of bleeding. The skin is then marked in this location using a permanent ink marker. A metal object (2 inch paper clip) is then placed over the localized bleeding site in order to identify the site during angiography. During the subsequent arteriogram the arterial supply to the bleeding site was easily localized if actively bleeding. However, when extravasations were not visualized on the arteriogram, the arterial supply was unique to the extravasations site and empiric embolization could be considered.

### Embolization technique

Superselection of the artery supplying the area of hemorrhage was performed using a 3-French microcatheter (Renegade, Boston Scientific, Natick, MA). This catheter was advanced coaxially to the bleeding site (marked by the clip) through the indwelling 4 or 5-French catheter. Attempts were made to position the catheter as close to the bleeding site as possible. Depending on the anatomy the catheter was either advanced through the superior mesenteric artery or inferior mesenteric artery distal branch (i.e. distal middle colic artery marginal artery). Embolization was then performed using 2.0–2.5 cc of 500–700 micron particles either Polyvinyl alcohol (Contour, Boston Scientific, Natick, Massachusetts, USA), Embospheres (Biosphere Medical, Rockland, Massachusetts, USA), or Bead Block Compressible Microspheres (Terumo Medical Systems (Tokyo, Japan). 2.0–2.5 cc of particles were used for each branch whether the bleeding site was angiographically visible or not with the goal of occluding the distal branch of the artery (marginal artery and vasa recta) close to the bleeding site.

## Results

(See Table 1)

**Summary of Results:** Summary of Results

Patient #	Age/Sex	Nuclear Medicine Source of Bleeding	Transfusion Requirment (Packed Red Cells Units)	Hgb level prior to transfusion g/dl	Time between marker placement and angiography	Angiographically positive	Hemostasis after embolization	Etiology of bleeding
1	70/M	Hepatic Flexure of Colon	5	11.4	< 2 hours	Yes	Yes	Diverticulosis

2	84/F	Hepatic Flexure of Colon	5	5.4	< 2 hours	No	Yes	Suspected diverticulosis

3	65/F	Splenic Flexure of Colon	5	7	< 2 hours	No	Yes	Unknown

4	55/F	Splenic Flexure of Colon	12	7.9	< 2 hours	No	Yes	Submucosal vascular ectasia

5	68/M	Rectum	7	11	< 2 hours, 18 hours for 2nd intervention	Yes, during 2nd intervention	Yes, after 2nd intervention	Rectal ulcer due to rectal tube

All five patients had cessation of bleeding following embolization, even if the site was angiographically not visible and empiric embolization was performed based on the site of the clip. One patient (#5) required a repeat angiogram and embolization before bleeding was stopped. This patient initially had empiric embolization of distal branches of the superior hemorrhoidal artery. Overnight the patient continued to bleed, so the next day a superselective middle hemorrhoidal arteriogram (from the anterior division of the internal iliac artery) demonstrated the bleeding site. This area was then embolized using the above described technique. Previous colonoscopy/sigmoidoscopy performed by an experienced gastroenterologist failed to provide a means to stop the bleeding in patient #5.

**Figure 1:**
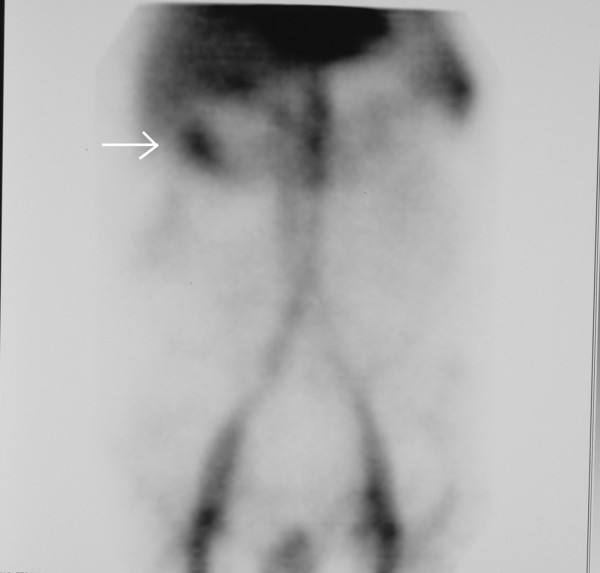
**Nuclear Medicine tagged red blood cell scan of patient #1 demonstrates focal extravasation from the hepatic flexure**. Arrow points to extravasation site.

**Figure 2:**
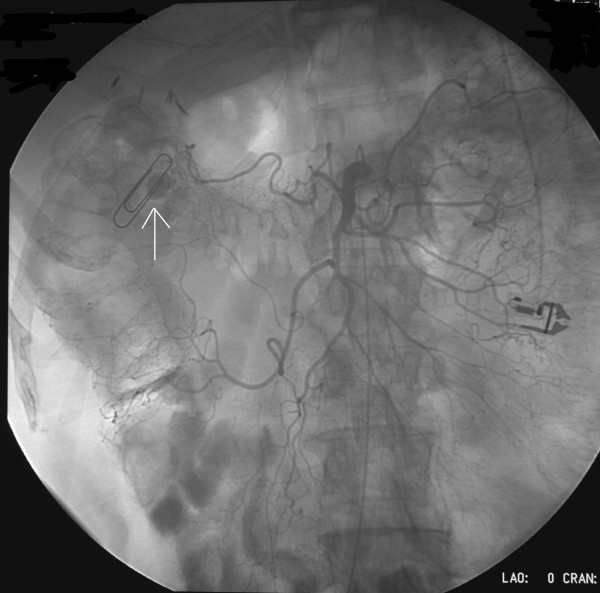
**Superior mesenteric arteriogram of patient #1 in the AP projection**. Note the right branch of the middle colic artery supplying the site of bleed (paper clip) based on nuclear medicine scan. Arrow points to paper clip and extravasation site.

**Figure 3:**
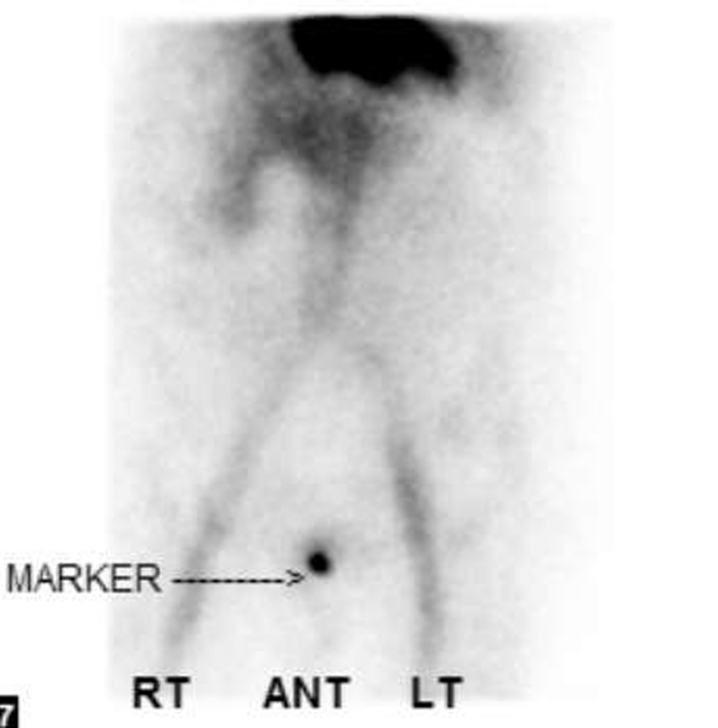
**Nuclear Medicine tagged red blood cell scan of patient #5 demonstrates focal extravasation from the rectum**. Marker denotes extravasation site.

**Figure 4:**
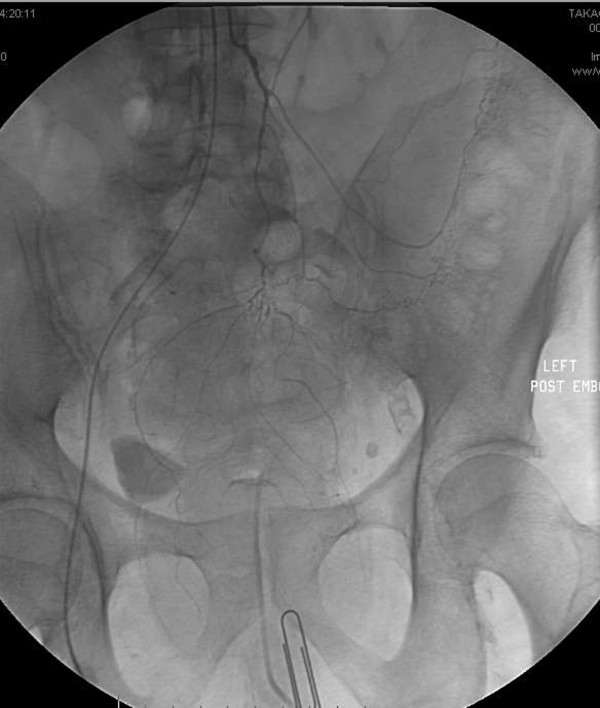
**Selective inferior mesenteric angiogram demonstrates no extravasation of from the branches of the superior hemorrhoidal artery with attention to the paper clip marker region**. These branches were selectively embolized empirically, but the patient continued to bleed overnight.

**Figure 5:**
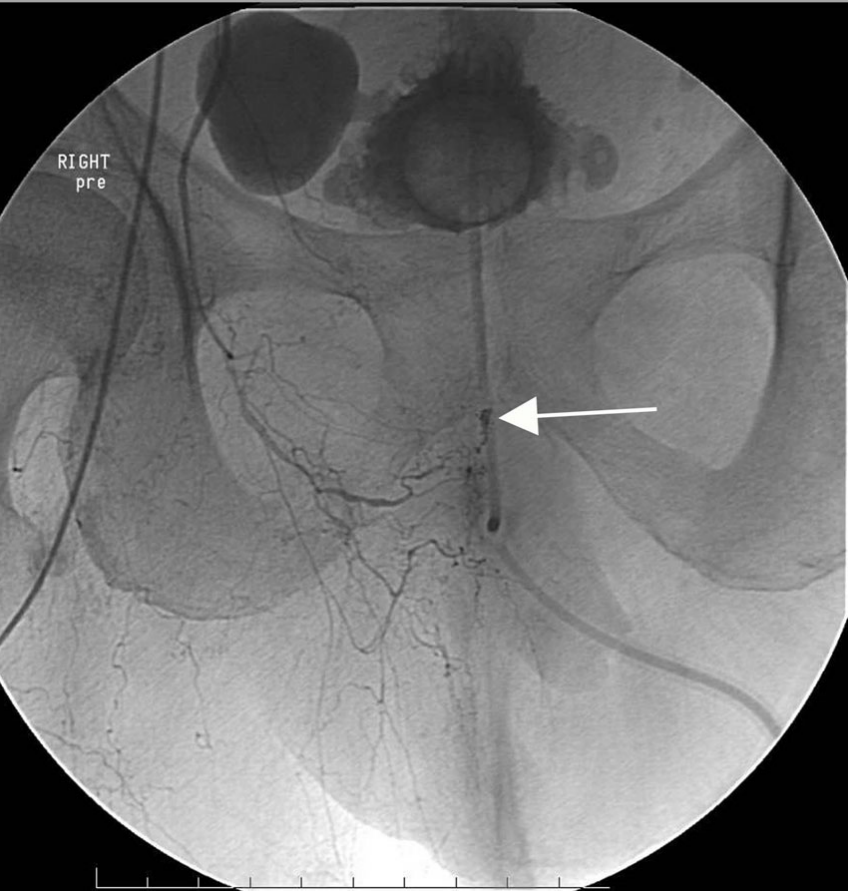
**Selective right middle hemorrhoidal angiogram demonstrates extravasation from a distal branch (arrow) in the vicinity of the paper clip marker that was present the day before**. This was embolized and bleeding stopped.

In 2 patients which the bleeding site was angiographically positive (patients #1 and #5) the placement of the clip helped direct appropriate superselection of the target artery (Figure 1, 2, 3, 4, 5). In one of these patients because the hemorrhage was intermittent angiographically, the clip allowed real time targeting of the appropriate hemorrhaging branch. These two patients prospectively demonstrated the surprising accuracy of the clip localization technique.

In 3 patients in which the bleeding site was angiographically negative even after superselection (patient #2, #3, and #4), the clip allowed empiric selective embolization of the artery supplying the area under the clip.

Follow up of 4 of these patients with colonoscopy demonstrated cessation of hemorrhage and no evidence of ischemia. Pathology on one patient (#4) following the patients demise demonstrated the gastrointestinal bleed was due to a vascular malformation in the splenic flexure of the colon described as submucosal vascular ectasia. A thrombosed bleeding point is seen histologically from the lesion. Vascular sclerosis was noted indicating appropriate target embolization. This patient expired 30 days following the procedure due to complications of ARDS/multiorgan failure secondary to Staphylococcus Aureus bronchopneumonia related sepsis.

## Discussion

Technetium-labeled red blood cells scintigraphy is noninvasive method of localizing lower gastrointestinal bleeding that can be performed at the bedside of critically ill patients. [2,3] The advantage of scintigraphy is that it is more sensitive (0.1 cc/minute) than angiography (0.5 cc/min). [4,5] The disadvantage of scintigraphy is that it can only localize to a general area of the intestine making anatomic localization less precise. This may be adequate for segmental resection, but is usually thought to be inadequate for catheter directed embolization.

On the other hand, catheter directed angiography can be both diagnostic and provide a means for therapy through embolization. An advantage of angiography is its precision in anatomic localization of a bleeding site or nonbleeding vascular abnormality. [6] However, the procedure cannot be performed at the bedside, has a risk of contrast induced nephrotoxicity and has minimal risk of contrast reaction. Angiography may be negative in approximately 50% of massive lower gastrointestinal bleeding. [7] Furthermore, angiography is less sensitive than technetium-labeled red blood cells scintigraphy.

CT angiography offers a less invasive method than catheter angiography, however its sensitivity is still less than nuclear medicine bleeding scan (0.1 ml/min for scintigraphy versus 0.35 ml/min for CT). [5] However scintigraphy is often unavailable after hours, whereas CT is usually available 24 hours a day. CT angiography does offer the advantage of more precise localization of the bleeding source. Furthermore, critically important ancillary findings may also be demonstrated on CT. In the cases above scintigraphy was utilized due to its greater sensitivity.

The concept of colonic embolization for lower gastrointestinal bleeding was first reported in 1977 by Goldberger and Bookstein. [8] In 1992, Guy et al reported the first series of microcatheter embolization for lower gastrointestinal bleeding. [9] The result showed that the superselective embolization procedure was successful in nine out of ten patients without any clinical evidence of intestinal infarction. In 1997, Gordon et al reported 17 additional cases of microcatheter embolization using microcoils, gelfoams, and polyvinyl alcohol particle without any clinically evidence of colonic infarction. [10] With advances in technology and refinement in technique, transcatheter embolization has demonstrated great promise as a primary modality in the management of acute lower gastrointestinal hemorrhage. [9-13]

Intra-arterial vasopressin infusion can also be effectively used to treat colonic bleeding. Vascopressin's clinical success has been quoted to be 83%–100% in colonic hemorrhage compared to 86%–100% for catheter directed embolization. Rebleeding rates for vasopressin infusion are high at 36%–43% versus 11%–19% for catheter directed embolization. Major complication rates for vasopressin were between 0%–21% and 9% of these were fatal. This compares to a major complication rate for modern catheter directed embolization of 1.3%. [14]

Current literature suggests that the use of microcoils may be superior to particles for embolization. Although we exclusively used particles for embolization in our series, the use of microcoils may offer a more precise alternative with less risk of ischemia. [15] However, in our cases where precise localization is not possible particles may provide greater area of distal embolization and the option of redo embolization if necessary.

A common problem however is the positive scintigram with negative angiography. In hemodynamically unstable patients, Ryan et al reported positive RBC scintigraphy with negative angiography in 31% of their patients (5 out of 16 patients). Similarly, in a nonrandomized series; Burgess et al reported this scenario in 27% of their patients (4 out of 15 patients). [16] In hemodynamically stable patients, Zink et al reported this scenario in 77.8% (14 out of 18 patients). [5] When vessels were embolized without the benefit of our technique as shown by Burgess et al there was an unfavorable outcome with two patients having proven ischemia and one having continued bleeding. [16]

Although some of these bleeds resolve spontaneously, there have been two approaches to solving this dilemma of persistent bleeding that have been previously described. These include provocative bleeding techniques and carbon dioxide arteriography. [17,18]

Provocative bleeding techniques (utilizing intrarterial heparin, tolazoline and urokinase) have been limited (with relatively small series) because of the theoretical risk of uncontrolled bleeding when either (1) during active bleeding when the site is not localized arteriographically and (2) can be visualized angiographically, but cannot technically embolized. In one series 6 out of 16 patients were provoked into bleeding. 5 of these patients had a positive red blood cell scan, but only 3 out of these were able to undergo catheter directed embolization. [19] In another series of 7 patients 2 out of 7 patients were able to be provoked into bleeding with resultant surgical repair of the bleeding site. [18] Therefore, provocative bleeding can be a useful tool in diagnosis of colonic bleeding in the setting of positive scintigraphy and negative angiography.

Carbon dioxide angiography is limited in patients who cannot suspend respiration and in patients who have excessive bowel gas motion. There have also been reports of bowel necrosis after hand delivery of carbon dioxide injection. [20]

We therefore present a simple technique to address this difficulty. This technique consists of a metal marker (paper clip) that is placed on the abdomen during the scintigraphic study over the site of active extravasation. Whether or not there is positive selective angiogram (showing extravasation) this marker is used to localize the culprit artery, thereby allowing consideration for embolization. This technique was accurate in our series. Furthermore, in all five attempted patients successful embolization and bleeding cessation occurred. There was no evidence of colonic ischemia or infarction in any of these patients, although the sample size is small. These patients were also spared the risks associated with surgery. This technique offers an alternative and complements the above mentioned techniques (provocation and CO2 angiography). The use of this clip marker technique does not preclude the use of the either provocative agents or carbon dioxide arteriography prior to embolization.

An endoscopic clip marker technique has been previously described in upper gastrointestinal bleeding to facilitate angiographic localization and embolization. [21] Our technique is helpful for localization in colonic bleeding. The technique is dependent on the unique anatomic configuration of the colon in the periphery of the abdomen where each segment of the colon is supplied by a relatively unique one or two end artery analogous to the spokes in a wheel. This situation is does not hold in the small bowel where due to redundancy and overlapping of the small bowel loops occurs, thereby limiting the use of this technique in this portion of the gastrointestinal tract. One potential problem of our technique is that due to colonic motility the paper clip localization will change. It is known that the colon is tethered at multiple points and therefore is limited in its ability to have major shifts in position, unlike the small bowel. [22] Also the likelihood of major displacement in colonic position is very low in the time span between nuclear medicine localization and angiography (usually within 1–2 hours).

One issue that arose during empiric embolization was the lack of a definite therapeutic endpoint. Our therapeutic endpoint was clinically based on restoration of hemodynamic stability that usually occurred within 15 minutes of adequate embolization. However, we realize that this is a shortcoming. We have overcome this by limiting our particulate volume to no more than 2.0–2.5 cc of the standard concentration of particles (500–700 μm) in the hopes of occluding only the vasa recta in the vicinity of our bleeding site. This is based on our experience with angiographically positive colonic bleeding sites (example Case #1). The reported risk of colonic ischemia in standard angiographically localized embolization is less than 10%. [23] We recognize that there is a higher theoretical risk of colonic ischemia using this technique compared to standard angiographically localized embolization. However, this risk is in the context of a life threatening situation in a potentially high surgical risk patient.

With rectal bleeding as in patient 5 it should be remembered that this area is supplied from both the internal iliac anterior division as well as the inferior mesenteric artery. [24] Therefore, consideration should be made for superselective angiography of both systems with the paper clip marker as a guide.

In summary, the dilemma of positive scintigraphic evidence of colonic bleeding with negative arteriography can be resolved with the use of a metal marker during the scintigram to guide superselective angiography. Though this technique is useful, it is merely designed to be an adjunct to the currently available modalities of treating colonic bleeding. Although in our small series of patients this technique appears to be simple, safe and effective, further clinical investigation is warranted with a larger patient population. In life threatening bleeding with positive scintigraphy and negative angiography even after superselection (as occurred in 3 of our patients) extreme caution should be utilized in embolization using the clip localization method. Though in our small series we had no complications this may have been fortuitous. In another series of 5 patients (Burgess et. al.) there was a high rate of colonic ischemia when embolization was performed based on positive scintigraphy alone with negative angiography. The rate of intestinal ischemia was 60% and the mortality from ischemia or uncontrolled bleeding was also 60%. [16] We realize that empiric embolization using this technique may be less precise than standard angiographically positive embolization. This is due to the lack of exact anatomic localization and a definite therapeutic endpoint. However, this technique may offer a role in therapy in coordination with the colorectal surgeon for the high risk patient in an otherwise life threatening situation.

## Competing interests

The authors declare that they have no competing interests.

## Authors' contributions

MIS: Performance of cases, writing and compiling of manuscript, review of literature, selection of figures.

AS: Review of literature, writing and compiling of manuscript and tables, editing and selection of figures.
